# FEV_1_ decline in relation to blood eosinophils and neutrophils in a population-based asthma cohort

**DOI:** 10.1016/j.waojou.2020.100110

**Published:** 2020-03-17

**Authors:** Helena Backman, Anne Lindberg, Linnea Hedman, Caroline Stridsman, Sven-Arne Jansson, Thomas Sandström, Bo Lundbäck, Eva Rönmark

**Affiliations:** a Dept of Public Health and Clinical Medicine, Section of Sustainable Health/the OLIN Unit, Umeå University, Umeå, Sweden; b Dept of Health Sciences, Luleå University of Technology, Luleå, Sweden; c Dept of Public Health and Clinical Medicine, Section of Medicine, Umeå University, Umeå, Sweden; d Krefting Research Centre, Institute of Medicine, University of Gothenburg, Gothenburg, Sweden

**Keywords:** Asthma, FEV_1_, Eosinophils, Neutrophils, Cohort, ANOVA, Analysis of variance, ATS, American Thoracic Society, BMI, Body mass index, ECRHS, European Community Respiratory Health Survey, EOS, Eosinophils, ERS, European Respiratory Society, FEV_1_, Forced Expiratory Volume in 1 s, FEV_1_pp, FEV_1_ percent of predicted, FVC, Forced Expiratory Volume, GLI, Global Lung function Initiative, ICS, Inhaled corticosteroids, IgE, Immunoglobulin E, L, Liters, Ml, Milliliters, N, Number, NEU, Neutrophils, OCS, Oral corticosteroids, OLIN, Obstructive Lung Disease in Northern Sweden, OLS, Ordinary Least Squares, VGDF, Vapors, gas, dust or fumes

## Abstract

**Background:**

The relationship between lung function decline and eosinophils and neutrophils has important therapeutic implications among asthmatics, but it has rarely been studied in large cohort studies.

**Objective:**

The aim is to study the relationship between blood eosinophils and neutrophils and FEV_1_ decline in a long-term follow-up of a population-based adult asthma cohort.

**Methods:**

In 2012–2014, an adult asthma cohort was invited to a follow-up including spirometry, blood sampling, and structured interviews, and n = 892 participated (55% women, mean age 59 y, 32–92 y). Blood eosinophils, neutrophils and FEV _1_ decline were analyzed both as continuous variables and divided into categories with different cut-offs. Regression models adjusted for smoking, exposure to vapors, gas, dust, or fumes (VGDF), use of inhaled and oral corticosteroids, and other possible confounders were utilized to analyze the relationship between eosinophils and neutrophils at follow-up and FEV_1_ decline.

**Results:**

The mean follow-up time was 18 years, and the mean FEV _1_ decline was 27 ml/year. The annual FEV_1_ decline was related to higher levels of both blood eosinophils and neutrophils at follow-up, but only the association with eosinophils remained when adjusted for confounders. Further, the association between FEV_1_ decline and eosinophils was stronger among those using ICS. With EOS <0.3 × 10^9^/L as reference, a more rapid decline in FEV1 was independently related to EOS ≥0.4 × 10^9^/L in adjusted analyses.

**Conclusions and clinical relevance:**

Besides emphasizing the importance of smoking cessation and reduction of other harmful exposures, our real-world results indicate that there is an independent relationship between blood eosinophils and FEV_1_ decline among adults with asthma.

## Introduction

It is well recognized that accelerated lung function decline is associated with adverse health outcomes including mortality.^1^ Impaired lung function is common among asthma patients, and a population-based study from the late 1990s showed that over 15 years, subjects with asthma had a larger mean annual FEV_1_ decline than subjects without asthma.^2^ In contrast, a more recent study from the European Community Respiratory Health Survey (ECRHS) found that the annual FEV_1_ decline was quite similar in adults with and without asthma.^3^ Although not all asthmatics experience accelerated FEV_1_ decline, it infers an increased burden of disease and mortality risk on those affected.

Large longitudinal population-based studies on lung function decline among asthmatics are uncommon, and most knowledge is currently based on cross-sectional results and on patients recruited from health care. Previous studies have shown associations between impaired lung function and smoking,^2^^,^^4^ occupational exposures,^5^^,^^6^ low initial FEV_1,_^7^ bronchial hyper-reactivity,^7^^,^^8^ atopy,^8^ age,^2^ duration and severity of disease,^7^^,^^9^ and airway inflammation.^10^, ^11^, ^12^

Several studies have shown associations between severe asthma and eosinophil and/or neutrophil counts in induced sputum,^13^ and reducing particularly eosinophilic inflammation is of importance in the management of asthma. However, there is no consensus on how to define increased levels of eosinophils or neutrophils, and the knowledge about cut-offs that associate with accelerated lung function decline among asthmatics is limited. Further, although we know that higher levels of sputum eosinophils and neutrophils are commonly associated with airway obstruction in asthma,^12^^,^^14^ the knowledge on the association with longitudinal changes in FEV_1_ is limited. And importantly, the relationship between eosinophil and neutrophil counts in blood and FEV_1_ decline is even less studied.

Therefore, we aimed to study the relationship between blood eosinophils and neutrophils and FEV_1_ decline in a long-term follow-up of a population-based adult asthma cohort.

## Methods

### The asthma cohort

The asthma cohort (n = 2055) consists of adults with asthma in northern Sweden identified at clinical examinations of population samples performed between 1986 and 2001 within the Obstructive Lung Disease in Northern Sweden (OLIN) studies. All subjects reporting physician-diagnosed asthma, those reporting ever having had asthma, and also those with a medical history of asthma along with physiologically verified bronchial variability or asthma medication were included, as previously described.^15^

In 2012–2014, all subjects in the asthma cohort still living in Norrbotten (n = 1425) were invited to a follow-up including a detailed structured interview, spirometry, blood sampling for total IgE, and blood cell counts, in which 1006 (71%) participated.^15^^,^^16^ The current study includes subjects with data on both lung function measurements at baseline and follow-up, as well as on blood cell counts (n = 892).

### Spirometry and FEV_1_ decline

At study entry, spirometry was performed according to guidelines of the European Respiratory Society (ERS) and American Thoracic Society (ATS),^17^^,^^18^ with a Mijnhardt Vicatest 5 dry volume spirometer. At the 2012–14 follow-up, spirometry was performed according to the 2005 ERS/ATS guidelines^17^ using a Jaeger Masterscope pneumotach spirometer, as previously described in detail.^15^^,^^16^ Lung function decline was assessed as annual change in pre-bronchodilator FEV_1_ in ml (FEV_1_ml) and FEV_1_ % of predicted^19^ (FEV_1_pp), respectively, between the first examination (in 1986–2001) and the follow-up. The annual change was calculated as the value at follow-up minus the value at the first examination divided by the number of years in-between. The annual change in FEV_1_pp was also divided by quartiles and rapid decline in FEV_1_ was defined as the lowest (most accelerated or rapid) 25% of the n = 892 values.

### Blood eosinophils and neutrophils

At follow-up, the absolute levels of eosinophil (EOS) and neutrophil (NEU) counts in blood were assessed both as continuous variables, and categorized into groups: EOS<0.3, 0.3≤EOS<0.4 and EOS≥0.4 × 10^9^/L, and NEU<4.0, 4.0≤NEU<5.0 and NEU≥5.0 × 10^9^/L, respectively.

### Statistical analyses

IBM SPSS Statistics 25.0 (Armonk, NY) was used for statistical analyses. In bivariate analyses, the Chi-square test or Mantel-Haenszel test for trend was used to test for differences in proportions and the T-test or ANOVA, as appropriate, were used to test for differences in means. The Spearman rho correlation coefficient was used to evaluate correlations between FEV1 decline and EOS and NEU. P-values <0.05 were considered statistically significant. In adjusted regression models, EOS, NEU, age and body mass index (BMI) at follow-up, sex, number of pack-years, exposure to vapors, gas, dust, or fumes (VGDF) at work, number of years of follow-up, allergic sensitization, FEV_1_ reversibility at study entry, FEV_1_ <80% at study entry, and inhaled (ICS) and oral corticosteroid (OCS) use at follow-up and/or at study entry were included as independent variables (see Supplementary variable definitions for more information). Linear regression (OLS) was performed with annual FEV_1_ decline as dependent variable and with EOS and NEU as continuous independent variables. Logistic regression models were performed with rapid decline in FEV_1_pp (the most rapid 25%) as outcome variable (non-rapid as reference), and with EOS and NEU included as categorical covariates.

Several sensitivity analyses are briefly presented in the results section, with more thorough information available as Supplementary material. Adjusted logistic regression analyses were performed with the outcome variable based on different definitions of rapid decline in FEV_1_. These definitions were based on annual decline in FEV_1_ assessed in ml and as Z-scores based on the OLIN and Global Lung function Initiative (GLI) reference values, respectively. These sensitivity analyses also include log-transformations of EOS and NEU and a comparison of rapid decline definitions divided by tertiles, quartiles, quintiles, sextiles, and septiles.

## Results

### Characteristics and mean annual FEV_1_ decline

Among the n = 892 asthmatics, the mean age at follow-up was 58.5 y (min-max 32–92 y), and 55% were women. While men were more often former smokers, women tended to be more often current smokers, both at study entry and at follow-up. Further, men had more pack years of smoking and had been more frequently exposed to vapors, gas, dust or fumes (VGDF) at work. At follow-up, a substantially larger proportion was using ICS compared to at study entry (Table 1), and ICS users had higher levels of eosinophils (Table 2). The mean annual FEV_1_ decline among all subjects was −27ml and −0.07 units in FEV_1_pp, respectively, and was higher among men, −32 ml (−0.18pp), than among women, −24 ml (0.02pp), (p < 0.001 for both FEV_1_ml and FEV_1_pp by sex), and among ever-smokers, −30 ml (−0.16pp), than among never-smokers, −25 ml (0.02pp) (p < 0.001 for both FEV_1_ml and FEV_1_pp by smoking habits), displayed divided by pack-years of smoking in Table 3.

**Table 1:** Characteristics at study entry and at follow-up, by sex and among all subjects.

Characteristics	At study entry (1986–2001)			At follow-up (2012–2014)		
	By sex			By sex		
	Women N = 495	Men N = 397	All N = 892	Women N = 495	Men N = 397	All N = 892
Mean (SD) pre-bronchodilator FEV_1_ in Liters	2.84 (0.55)	3.73 (0.82)	3.24 (0.81)	2.40 (0.63)	3.12 (0.84)	2.72 (0.81)
Mean (SD) pre-bronchodilator FEV_1_% of predicted	90.2 (12.5)	86.6 (14.9)	88.6 (13.8)	90.3 (15.4)	83.1 (16.4)	87.1 (16.2)
Mean (SD) number of years of follow-up				18.2 (4.2)	18.7 (4.4)	18.4 (4.3)
Mean (SD) age (years)	39.6 (11.7)	40.6 (11.1)	40.0 (11.5)	57.9 (12.5)	59.3 (11.7)	58.5 (12.2)
Min-max age (years)	19–70	19–69	19–70	33–92	32–92	32–92
Mean (SD) BMI	25.1 (4.6)	26.1 (3.4)	25.6 (4.1)	28.2 (5.4)	28.9 (4.4)	28.5 (5.0)
Family history of asthma	44.8%	36.8%	41.3%			
Allergic sensitization		N/A		26.8%	42.9%	33.9%
OCS use last 12 months		N/A		2.2%	1.3%	1.7%
ICS use last 12 months	10.9%	12.8%	11.8%	48.1%	38.3%	43.7%
Never-smoker	47.1%	43.1%	45.3%	50.3%	46.3%	48.5%
Former smoker	25.1%	31.7%	28.0%	36.4%	43.3%	39.5%
Current smoker	27.9%	25.2%	26.7%	13.3%	10.3%	12.0%
Mean (SD) number of pack years of smoking^a^		N/A		14.3 (14.2)	21.3 (17.3)	17.5 (16.0)
VGDF exposure		N/A		21.0%	62.9%	39.7%

Data presented as column % unless otherwise stated, Allergic sensitization = Positive on Phadiatop (>0.35 kU/L), SD = Standard deviation.

VGDF = vapors, gas, dust or fumes, L = liters, pp = %of predicted, BMI = body Mass Index.

N/A = Data not available, ICS = Inhaled corticosteroid, OCS = Oral corticosteroid.

a Among former and current smokers

**Table 2:** Lung function and proportions with blood eosinophils (EOS) < 0.3, 0.3 ≤ EOS<0.4, and EOS≥0.4 × 109/L, respectively, by the use of inhaled corticosteroids (ICS) at follow-up.

		ICS use at follow-up		p-value^a^
		ICS naive	ICS users	
		n = 502	n = 390	
*Blood eosinophils at follow-up*				
EOS<0.3	n (%)	394 (78.5)	255 (65.4)	
0.3 ≤ EOS<0.4	n (%)	52 (10.4%)	62 (15.9)	
EOS≥0.4	n (%)	56 (11.2)	73 (18.7)	**<0.001**
*Lung function*				
FEV1 % of predicted at baseline	mean (SD)	91.0 (12.3)	85.6 (14.9)	**<0.001**
FEV1 % of predicted at follow-up	mean (SD)	89.1 (14.9)	84.4 (17.5)	**<0.001**
Annual FEV1 decline in % of predicted	mean (SD)	−0.09 (0.55)	−0.05 (0.69)	0.384
Annual FEV1 decline in ml	mean (SD)	−28.2 (19.2)	−26.5 (23.7)	0.239

a chi-square p-value for difference in proportions or T-test p-value for difference in means between groups based on ICS use at follow-up. EOS = blood eosinophils at follow-up, ICS = Inhaled corticosteroids at follow-up. Bold values indicate statistical significance

**Table 3:** Mean cell counts in blood (10^9^/L) at follow-up and mean annual decline in FEV_1_ by smoking habits.

		Mean cell count in blood		Mean annual decline	
		Eosinophils	Neutrophils	FEV1pp	FEV1ml
Never-smoker (N = 433)	Mean (SD)	0.21 (0.17)	3.57 (1.25)	0.02 (0.58)	−24.6 (20.8)
<10 PY (N = 170)	Mean (SD)	0.21 (0.16)	3.71 (1.39)	−0.01 (0.58)	−25.3 (20.3)
10 ≤ PY < 20 (N = 124)	Mean (SD)	0.23 (0.17)	3.91 (1.44)	−0.16 (0.65)	−28.9 (21.7)
20 ≤ PY < 30 (N = 86)	Mean (SD)	0.22 (0.15)	4.21 (1.70)	−0.28 (0.65)	−34.4 (22.1)
PY ≥ 30 (N = 79)	Mean (SD)	0.22 (0.15)	4.37 (1.48)	−0.33 (0.62)	−37.7 (19.7)
ANOVA	P-value	0.639	**<0.001**	**<0.001**	**<0.001**

PY = Packyears of smoking at follow-up, FEV_1_pp = FEV_1_ percent (%) of predicted, SD = Standard deviation, Bold values indicate statistical significance.

A mean annual decline in FEV_1_pp of e.g. −0.33 implicates an average decrease of 1 unit in FEV_1_% pf predicted over three years, e.g. from 73% to 72%. A mean annual decline in FEV_1_ml of e.g. −33 ml implicates an average decrease of about 100 units in FEV_1_ml over three years, e.g. from 3200 ml to 3100 ml

### Relationship between blood eosinophils and annual FEV_1_ decline

**Fig. 1:**
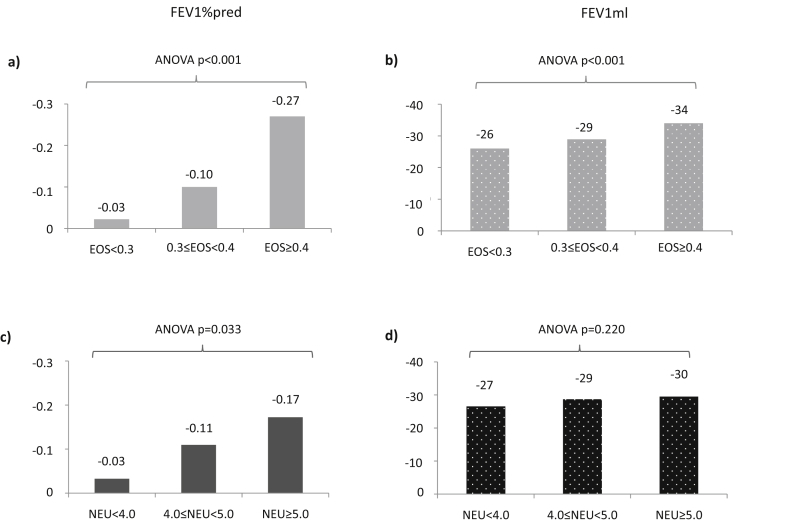
a–d. Mean annual change in FEV1% of predicted (FEV1%) and FEV1 in ml (FEV1ml) within categories of blood eosinophils (a and b) and neutrophils (c and d) at follow-up, respectively. P-values from ANOVA for test of differences between groups

**Fig. 2:**
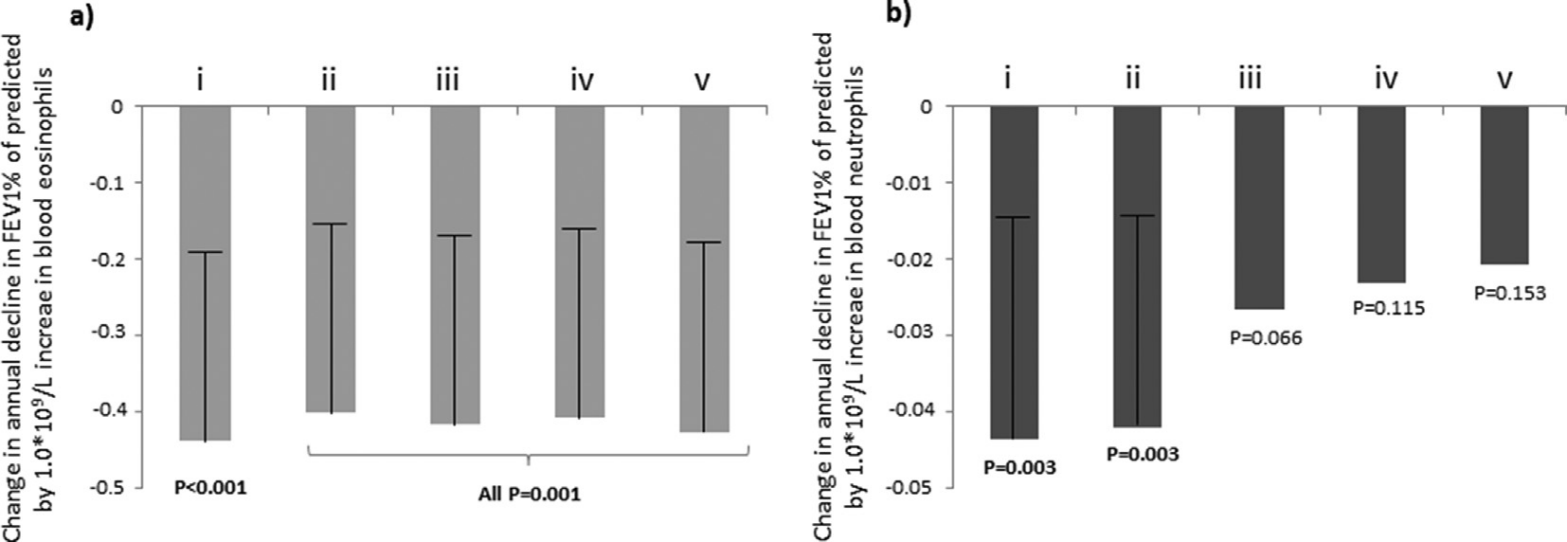
a–b. Mean annual change in FEV1% of predicted by one unit change in absolute levels (1.0∗10^9^/L) of blood eosinophils (2a) and neutrophils (2b) at follow-up, respectively, assessed by B-coefficients with upper 95% CI and corresponding p-values from linear regression models. (i) = Unadjusted, (ii) = Adjusted for eosinophils (in Fig. 2b) and neutrophils (in Fig. 2a) in blood, number of years of follow-up, age, height and sex, (iii) = Adjusted for the same as (ii) and also for number of packyears of smoking and exposure to vapors, gas, dust or fumes at work, (iv) = Adjusted for the same as (iii) but also for BMI and allergic sensitization, (v) = Adjusted for the same as (iv) but also for ICS use and OCS use at study entry and/or follow-up, FEV1<80% of predicted at study entry, and significant FEV1 reversibility at study entry

The mean annual FEV_1_ decline, both in terms of FEV_1_pp and FEV_1_ml, was significantly larger among subjects with higher levels of EOS (Fig. 1a and b). The Spearman rho correlation coefficient for association between annual FEV_1_pp decline and EOS was −0.123 (p < 0.001), with stronger association when only including those using ICS at follow-up, i.e. −0.203 (p < 0.001). The association between EOS and FEV_1_ decline remained significant also in linear regression models adjusted for NEU and possible confounders (Fig. 2a and Supplementary Figure E1).

### Relationship between blood neutrophils and annual FEV_1_ decline

The mean annual decline in FEV_1_pp was significantly larger among subjects with higher levels of NEU, while the mean annual decline in FEV_1_ml was not (Fig. 1c and d). The Spearman rho correlation coefficients for the association between annual FEV_1_pp decline and NEU was −0.113 (p = 0.001), and −0.127 (p = 0.012) when only including those using ICS at follow-up. There was, however, no significant association between NEU and FEV_1_ decline when adjusted for EOS, smoking, exposure to VGDF, and other possible confounders in the linear regression models (Fig. 2b and Supplementary Figure E2).

### Blood eosinophils and neutrophils in relation to rapid decline in FEV_1_

Among subjects with EOS <0.3, 0.3 ≤ EOS < 0.4, and EOS ≥0.4, respectively, 21.4%, 28.9%, and 39.5% (p < 0.001) had rapid FEV_1_ decline. Among subjects with NEU <4.0, 4.0 ≤ NEU < 5.0, and NEU ≥5.0, respectively, 22.0%, 30.2%, and 29.8% (p = 0.014) had rapid FEV_1_ decline.

**Fig. 3:**
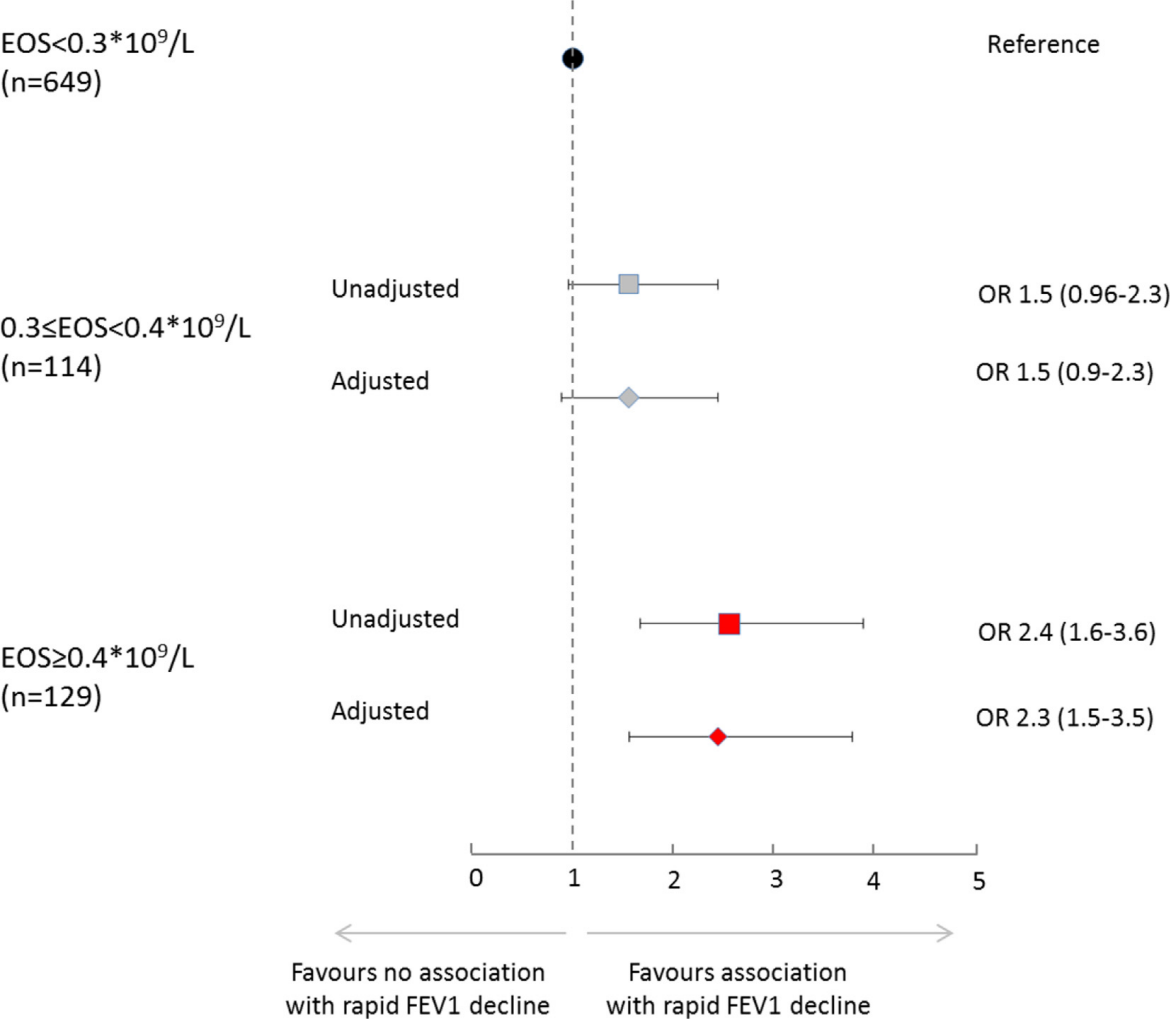
Unadjusted and adjusted associations between categories of blood eosinophils at follow-up and rapid decline in FEV1 (annual decline of −0.44 units or more in FEV1 % of predicted), expressed as odds ratios (OR) with 95% CI from logistic regression models (EOS<0.3∗10^9^/L as reference). The outcome variable was rapid decline in FEV1% of predicted defined as the lowest (most rapid) 25% of all values (non-rapid decline as reference). Adjusted = adjusted for categories of neutrophils in blood, number of years of follow-up, age, height, sex, number of pack years of smoking, BMI, allergic sensitization, exposure to vapors, gas, dust or fumes at work, ICS use and OCS use at study entry and/or follow-up, FEV1<80% of predicted at study entry, and categories of significant FEV1 reversibility at study entry in logistic regression models.

With subjects having EOS <0.3 as reference category, the odds ratios for rapid decline in FEV_1_ were 1.5 (95%CI 0.9–2.3) for subjects with 0.3 ≤ EOS < 0.4, and 2.3 (95%CI 1.5–3.5) for subjects with EOS ≥0.4, when adjusted for categories of NEU and possible confounders (Fig. 3). In the same logistic regression model, the odds ratios for rapid decline in FEV_1_ were 1.3 (95%CI 0.9–2.0) for subjects with 4.0 ≤ NEU < 5.0, and 1.2 (95%CI 0.7–1.8) for subjects with NEU ≥5.0, with subjects having NEU <4.0 as reference.

### Sensitivity analyses

Linear regression analyses stratified by smoking habits confirmed the main findings of an association between EOS and annual FEV_1_ decline among both never-smokers and ever-smokers, and the lack of association with NEU when adjusted (Supplemental Tables E1 and E2). Linear regression analyses stratified by sex revealed that, when adjusted, annual FEV_1_ decline was associated with EOS but not with NEU among women, while the opposite was observed among men (Supplemental Tables E3 and E4). Linear regression analyses with EOS and NEU entered into the model as log-transformed continuous independent variables confirmed the main findings (Supplemental Table E5).

The sensitivity analyses all confirmed the association with EOS≥0.4, and some results were significant also for 0.3 ≤ EOS < 0.4, while none of the NEU categories yielded statistically significant odds ratios in adjusted analyses (Supplemental Tables E6-E9). Further, analyses of the EOS cut-off of 0.150 did not yield significant associations with annual FEV_1_ decline (Supplemental Table E10).

## Discussion

The main results of this study were that FEV_1_ decline was related to both eosinophils and neutrophils in blood in this adult population-based asthma cohort study, but only blood eosinophils remained independently associated with FEV_1_ decline also when taking age, sex, smoking, BMI, corticosteroid use, initial FEV_1_ level, and other factors possibly related to FEV_1_ into account. Further, blood eosinophil counts ≥0.4∗10^9^/L was consistently and independently associated with larger decline in FEV_1_.

In the ECRHS starting in 1991–1993,^3^ the FEV_1_ decline was 29 ml/year over 9 years among n = 2116 subjects 20–56 years of age with asthma, results almost identical to our results of 27 ml/year. In 2 older studies, the decline was slightly higher.^2^^,^^20^ In the Australian Busselton Health Study performed between 1966 and 1995,^20^ the annual FEV_1_ decline among n = 1301 never-smoking adults with asthma was 28 ml/year among women and 40 ml/year among men, and in the Danish Copenhagen City Heart Study,^2^ the mean FEV_1_ decline was 38 ml/year among n = 1095 adults with asthma followed from 1976 to 1994. The less decline in more recent studies may be related to improvements in the treatment of asthma. However, neither of these two large population-based studies analyzed eosinophilic or neutrophilic inflammation in relation to FEV_1_ decline.

Our results of 27 ml annual FEV_1_ decline are also quite similar to those from smaller patient-based studies: 16.1–21.5 ml among n = 122 asthma patients on ICS treatment,^21^ 31.5 ml among frequent exacerbators vs 15 ml/year among asthmatics not defined as frequent exacerbators,^8^ 36 ml/year (0.34 pp/year) among those with <10packyears and 54 ml/year (0.75 pp/year) among those with ≥ 10 packyears among n = 203 Finnish asthmatics,^4^ and 51 ml in a study of n = 71 Dutch asthmatics completing a 2-year randomized controlled intervention study with bronchodilators.^8^ Blood eosinophils are related to severe asthma exacerbations,^22^ and it is possible that severe exacerbations contribute to excess FEV_1_ decline also in our study. Unfortunately, exacerbation data was lacking, why this could not be explored.

Airway eosinophilia is frequently observed in asthma but it is neither necessary nor sufficient for the development of the disease. However, the presence of eosinophils in sputum and blood can identify responders to inhaled or oral corticosteroids,^23^, ^24^, ^25^ and they are an important marker in the management of asthma. The ICS users also had higher levels of eosinophils, and lower FEV_1_ already at baseline. Interestingly, the association between FEV_1_ decline and blood eosinophils was stronger among those using ICS, results possibly due to confounding by indication, as prescriptions of ICS are more common in patients with a more severe asthma compared with those having mild asthma.^26^ It is well known from a large amount of clinical trials and also from population based studies^27^^,^^28^ that ICS use can prevent lung function decline in asthma patients. Thus, our results may underestimate the association between increased levels of EOS and decline in lung function.

Studies on random population-samples analyzing the relationship between lung function decline and blood cell counts have been lacking, but the association between eosinophils in blood and FEV1 decline among asthmatics was recently investigated in the Dunedin birth cohort^29^ which has been studied repeatedly from age 21 to 38. They found blood eosinophils significantly associated with decline in both the FEV_1_/FVC ratio and FEV_1_pp both among subjects with and without asthma,^29^ independent of smoking, which is well in line with our results. Neutrophil counts were, however, not analyzed in this study.

Although eosinophilia has been related to impaired lung function, all asthma patients with eosinophilia will not experience excess FEV_1_ decline,^10^^,^^12^ results that suggest complexity. Among n = 87 severe asthma patients in the UK with <10 pack-years of smoking and a mean FEV_1_ annual decline of 25.7 ml/year, it was actually the over-time variability in sputum eosinophilia rather than the baseline or follow-up eosinophil levels which associated with excess FEV_1_ decline.^12^ In contrast, one recent study on asthma patients showed that it was low (EOS <0.21 × 10^9^/L) rather than high baseline eosinophils that associated with excess FEV_1_ decline,^30^ and yet another study on n = 141 patients with adult onset asthma showed that neither blood nor sputum eosinophilia associated with accelerated FEV_1_ decline.^31^ Thus, it is not clear how eosinophilic inflammation affects lung function decline and more and larger studies seem required to disentangle this relationship among asthmatics, where our large population-based asthma cohort contributes with important information.

There is evidence that neutrophils in a large proportion of patients with neutrophilic asthma can be in an activated state, thus likely to cause tissue damage,^32^ and that these patients may also be unresponsive to corticosteroids.^33^ Correspondingly, we have previously shown that the levels of blood neutrophils are elevated among those with severe asthma in our cohort.^34^ Neutrophilic asthma may represent a phenotype associated with exposures such as cigarette smoking^32^^,^^35^ and excess lung function decline,^4^ which also would be in line with previous results showing that smoking is a risk factor for the development of chronic airway obstruction among asthmatics.^16^ In our study, men were much more frequently exposed to both smoking and VGDF than women. In the sex-stratified analyses, the relationship with FEV_1_ decline was consistently related to eosinophils among women. In contrast, neutrophils were related to lung function decline among men also after adjustment for confounders, which despite our efforts to account for exposures may be due to residual confounding caused by their more dominant and extensive exposure pattern. In summary, it seems that the relationship between lung function decline and eosinophils among asthmatics is much less related to external factors such as smoking and exposure to VGDF compared to the relationship between lung function decline and neutrophils. However, blood neutrophils are no precise predictors of sputum neutrophils,^35^ why there may exist associations between neutrophilic airway inflammation and FEV_1_ decline not detected in our study.

Different relevant cut-offs for defining increased eosinophils and neutrophils have been proposed, but so far there is no consensus. We found that subjects with blood eosinophil counts ≥0.4 × 10^9^/L consistently had an elevated risk for a larger decline in FEV_1_, in line with the results from the Dunedin study.^29^ Further, subjects with eosinophils in blood between 0.3 and 0.4 × 10^9^/L also had a significantly increased risk for a larger FEV_1_ decline in our sensitivity analyses. Different cut-offs have also been analyzed for associations between eosinophils and asthma exacerbations, e.g. >0.15, >0.30, and >0.50 × 10^9^/L,^36^ and different cut-offs for increased levels have been defined also for neutrophils.^32^ Further, blood eosinophils can predict sputum eosinophilia among asthmatics^35^^,^^37^ while blood neutrophils are more poorly related to sputum neutrophils,^35^ and interpreting and comparing different cut-offs in blood and sputum may be difficult. Results also based on induced sputum would have been valuable in the current study in order to assess inflammation in the target organs, i.e. the lungs. However, in contrast to analyses of induced sputum, analyses of cell counts in peripheral blood are suitable for large-scale studies due to methodological and cost-related matters, and as at least regarding eosinophils the correlation between blood and induced sputum is high.^35^^,^^37^

It has long been argued that airway inflammation is associated with airway re-modelling with thickening of the epithelium and sub-epithelial structures including the smooth muscles, all contributing to obstruction of the airway.^38^ The normal rate of lung function decline may be related to loss of elastic recoil in the lung and weaker diaphragm, as well as the inevitable life-long effects of exposure to pollutions and airway infections caused by e.g. viruses. Thus, the mechanism behind lung function decline due to ageing of the lung is multifactorial.

Regarding limitations of our study, we have no data on blood cell counts at baseline or during the follow-up period, why we cannot assess variability over time in e.g. eosinophils which previously has been associated with FEV_1_ decline when analyzed in sputum.^12^ Another possible limitation of this study is that as the FEV_1_ decline estimates were based on pre-bronchodilator values, the reversible obstruction aspect was not evaluated regarding the estimates of decline. However, analyses in the subsample with available post-bronchodilator values showed almost identical results (Supplementary material). An acute allergic inflammation at any time point could possibly be associated with both transient FEV_1_ decline and increased blood eosinophilia, however, as the individuals were not allowed to have a recent asthma exacerbation at the time of the examination, this is most probably not a source of bias. Also, different types of spirometers were used at study entry and at follow-up, which could affect the estimates of decline. However, this will affect all subjects in a similar way and thus probably not bias the associations with blood cell counts. With regards to strengths of this study, it is based on a large well-characterized population-based asthma cohort,^15^ enabling stratified analyses with sufficient power, and has a long follow-up time period. Our results on associations between FEV_1_ decline and eosinophils were clear and consistent across several different definitions. Further, the similarity of the mean annual FEV_1_ decline in our study with results from the few other large population-based studies on adults with asthma^2^^,^^3^^,^^20^ strengthens the external validity of our results.

Finally, in contrast to the current study, most previous large population studies on the association between FEV_1_ and blood cell counts are cross-sectional. It should be acknowledged that the long follow-up period probably reduces the effect of the day-by-day variability in FEV_1_ on the estimates of decline. Thus, this study provides novel data on progressing lung function changes among adults with asthma. Although baseline blood cell counts would have been of great value to assess prognostic associations, we still argue that the presented real-world data on a consistent relationship between FEV_1_ decline and blood eosinophils adds important input to the current body of evidence. Importantly, further research on asthma among adults is warranted, as we still have not seen an end to the increasing prevalence.^39^

In conclusion, this long-term follow-up of a population-based cohort of adults with asthma shows an association between FEV_1_ decline and blood eosinophils, independent of other factors and exposures. In contrast, the association between FEV_1_ decline and blood neutrophils was related to exposures such as smoking and vapors, gas, dust, or fumes at the work place. Besides emphasizing the importance of smoking cessation and reducing other harmful exposures, our results of a relationship between blood eosinophils and FEV_1_ decline among adults with asthma highlight both possibilities and need for other interventions.

## Ethics approval and consent to participate

All participants provided written informed consent, and ethical approval was achieved from the Regional Ethical Review Board at Umeå University (Dnr 2011-106-31M).

## Consent for publication

Not applicable.

## Availability of data and materials

The data that support the findings of this study are available from the corresponding author upon reasonable request.

## Funding

Supported by grants from The Swedish Heart & Lung Foundation, The Swedish Research Council, a regional agreement between Umeå University and Västerbotten County Council (ALF), Norrbotten County Council, the Swedish Asthma-Allergy Foundation, and VISARE NORR Fund: Northern county councils Regional federation. Additional support was provided by ThermoFisher, Uppsala, Sweden.

## Authors’ contributions

BL and ER designed the methodology for the longitudinal design of the asthma cohort. ER and HB designed the current study, interpreted the data, and drafted the manuscript. HB performed the statistical analyses. All authors contributed with interpretation of data, revised the manuscript critically for important intellectual content, and approved the final version to be submitted. All authors are accountable for all aspects of the work and ensure that questions related to the accuracy or integrity of any part of the work are appropriately investigated and resolved.

## Declaration of Competing Interest

Dr. Backman reports personal fees from Boehringer Ingelheim and AstraZeneca outside the submitted work, Dr. Lindberg reports personal fees from Boehringer Ingelheim, AstraZeneca, Novartis and Active Care outside the submitted work, Dr. Lundbäck reports grants from AstraZeneca, grants from GSK, personal fees from AstraZeneca, personal fees from GSK, and personal fees from Novartis, all outside the submitted work, Dr, Sandström, Dr Hedman and Dr Jansson have nothing to disclose, Dr Stridsman reports personal fees from Novartis and Astra Zeneca, outside the submitted work, and Dr. Rönmark reports grants from Astra Zeneca, grants from GlaxoSmithCline, outside the submitted work.
