# Supplementary material for: FEV1 decline in relation to blood eosinophils and neutrophils in a population-based asthma cohort
Source: World Allergy Organ J. 2020 Mar 17;13(3):100110. doi: 10.1016/j.waojou.2020.100110 (PMC7082214; doi:10.1016/j.waojou.2020.100110)
Supplement: Multimedia component 1 [file mmc1.docx]

**FEV1 decline in relation to blood eosinophils and neutrophils**

**in a population-based asthma cohort**

**Helena Backman, Anne Lindberg, Linnea Hedman, Caroline Stridsman, Thomas Sandström, Bo Lundbäck, Eva Rönmark**

**Online Data Supplement**

**Supplemental material: Definitions of factors included as covariates/independent variables in the regression models**

**Sex** was included as a dichotomous variable with men as reference.

**Age** was assessed both at study entry and at follow-up. Age at follow-up was included in the regression models.

**Number of years of follow-up** was calculated as the difference between the date at follow-up and the date at study entry.

**Allergic sensitization** for airborne allergens was defined as positive Phadiatop (>0.35kU/L) at follow-up. This variable included three categories: No (unexposed, used as reference category), Yes (exposed), and Not tested.

**Occupational exposure to vapours, gas, dust or fumes (VGDF)** at follow-up was defined as affirmative answer to the question “Have you been heavily exposed to vapours, gas, dust or fumes at work?”. This variable included three categories: No (unexposed, used as reference category), Yes (exposed), and Missing data.

**Smoking habits** were categorized as current smokers, ex-smokers, or non-smokers, and the number of pack-years among ever-smokers (ex- and current smokers at follow-up) was calculated by multiplying the number of packs of cigarettes smoked per day by the number of years of smoking.

**Significant FEV1 reversibility at study entry** was defined as change in FEV1 of >=12% and >=200ml after bronchodilation, and categorized into three groups: Negative (used as reference category), Positive, and Not tested (subjects with both FEV1/VC>0.7 and FEV1>90% of predicted were not tested for reversibility at study entry).

**FEV1<80% of predicted** value at study entry was based on the OLIN reference values and categorized as a dichotomous variable (0=No, 1=Yes).

**Inhaled corticosteroid (ICS) use** was assessed both at study entry and at follow-up, and was categorized into four groups: No ICS use (--), ICS use at study entry but not follow-up (+-), ICS use at follow-up but not study entry (-+), and ICS use at both study entry and follow-up (++).

**Oral corticosteroid (OCS)** use was assessed at follow-up and categorized as a dichotomous variable.

**Body Mass Index (BMI)** was assessed at both study entry and follow-up. In the regression models, BMI at follow-up was used.

**Table E1. Mean annual change in FEV1% of predicted by one unit change in absolute levels (1.0*10^9^/L) of eosinophils (EOS) and neutrophils (NEU) in blood, assessed by B-coefficients with 95% CI and corresponding p-values from linear regression models, among ever-smokers and never-smokers, respectively**

|  |  | **Among ever-smokers (n=459)** | | | | |  | **Among never-smokers (n=433)** | | | | |
| --- | --- | --- | --- | --- | --- | --- | --- | --- | --- | --- | --- | --- |
|  |  | **B** | **(95% CI)** | | | **P-value** |  | **B** | **(95% CI)** | | | **P-value** |
|  |  |  |  |  |  |  |  |  |  |  |  |  |
| Unadjusted | NEU | -0.035 | (-0.074 | - | 0.003) | 0.072 |  | -0.035 | (-0.079 | - | 0.009) | 0.116 |
|  | EOS | -0.479 | (-0.838 | - | -0.120) | 0.009 |  | -0.361 | (-0.679 | - | -0.043) | 0.026 |
|  |  |  |  |  |  |  |  |  |  |  |  |  |
| Adjusted A) | NEU | -0.033 | (-0.071 | - | 0.005) | 0.086 |  | -0.046 | (-0.089 | - | -0.002) | 0.039 |
|  | EOS | -0.416 | (-0.777 | - | -0.055) | 0.024 |  | -0.419 | (-0.736 | - | -0.103) | 0.010 |
|  |  |  |  |  |  |  |  |  |  |  |  |  |
| Adjusted B) | NEU | -0.025 | (-0.062 | - | 0.013) | 0.198 |  | -0.037 | (-0.082 | - | 0.007) | 0.100 |
|  | EOS | -0.427 | -0.788 | - | -0.067) | 0.020 |  | -0.442 | (-0.767 | - | -0.117) | 0.008 |
|  |  |  |  |  |  |  |  |  |  |  |  |  |
| Adjusted C) | NEU | -0.024 | (-0.062 | - | 0.014) | 0.213 |  | -0.028 | (-0.073 | - | 0.017) | 0.221 |
|  | EOS | -0.439 | (-0.806 | - | -0.072) | 0.019 |  | -0.403 | (-0.731 | - | -0.075) | 0.016 |
|  |  |  |  |  |  |  |  |  |  |  |  |  |
| Adjusted D) | NEU | -0.023 | (-0.061 | - | 0.015) | 0.238 |  | -0.021 | (-0.065 | - | 0.023) | 0.353 |
|  | EOS | -0.410 | (-0.780 | - | -0.040) | 0.030 |  | -0.476 | (-0.801 | - | -0.150) | 0.004 |
|  |  |  |  |  |  |  |  |  |  |  |  |  |

A) Adjusted for EOS, NEU, age, height, sex, no of years of follow-up. B) Adjusted for EOS, NEU, age, height, sex, no of years of follow-up, packyears of smoking at follow-up (among ever-smokers), occupational exposure to VGDF at follow-up. C) Adjusted for EOS, NEU, age, height, sex, no of years of follow-up, packyears of smoking at follow-up (among ever-smokers), occupational exposure to VGDF at follow-up, BMI, allergic sensitization. D) Adjusted for EOS, NEU, age, height, sex, no of years of follow-up, packyears of smoking at follow-up (among ever-smokers), occupational exposure to VGDF at follow-up, BMI, allergic sensitization, plus ICS use and OCS use, respectively, at both study entry and follow-up, FEV1<80% at study entry, and positive reversibility at study entry. Green color indicates statistical significance.

**Table E2. Mean annual change in FEV1ml by one unit change in absolute levels (1.0*10^9^/L) of eosinophils (EOS) and neutrophils (NEU) in blood, assessed by B-coefficients with 95% CI and corresponding p-values from linear regression models, among ever-smokers and never-smokers, respectively**

|  |  | **Among ever-smokers (n=459)** | | | | |  | **Among never-smokers (n=433)** | | | | |
| --- | --- | --- | --- | --- | --- | --- | --- | --- | --- | --- | --- | --- |
|  |  | **B** | **(95% CI)** | | | **P-value** |  | **B** | **(95% CI** | | | **P-value** |
|  |  |  |  |  |  |  |  |  |  |  |  |  |
| Unadjusted | NEU | -0.731 | (-2.039 | - | 0.577) | 0.273 |  | -1.161 | (-2.734 | - | 0.412) | 0.148 |
|  | EOS | -15.701 | (-27.925 | - | -3.477) | 0.012 |  | -13.948 | (-25.349 | - | -2.548) | 0.017 |
|  |  |  |  |  |  |  |  |  |  |  |  |  |
| Adjusted A) | NEU | -0.768 | (-2.039 | - | 0.503) | 0.235 |  | -1.467 | (-2.983 | - | 0.049) | 0.058 |
|  | EOS | -8.872 | (-21.048 | - | 3.304) | 0.153 |  | -10.292 | (-21.378 | - | 0.793) | 0.069 |
|  |  |  |  |  |  |  |  |  |  |  |  |  |
| Adjusted B) | NEU | -0.489 | (-1.769 | - | 0.790) | 0.453 |  | -1.133 | (-2.689 | - | 0.423) | 0.153 |
|  | EOS | -10.424 | (-22.711 | - | 1.863) | 0.096 |  | -11.407 | (-22.749 | - | -0.064) | 0.049 |
|  |  |  |  |  |  |  |  |  |  |  |  |  |
| Adjusted C) | NEU | -0.446 | (-1.737 | - | 0.845) | 0.498 |  | -0.734 | (-2.294 | - | 0.826) | 0.356 |
|  | EOS | -11.163 | (-23.673 | - | 1.348) | 0.080 |  | -9.515 | (-20.881 | - | 1.851) | 0.101 |
|  |  |  |  |  |  |  |  |  |  |  |  |  |
| Adjusted D) | NEU | -0.482 | (-1.751 | - | 0.788) | 0.456 |  | -0.331 | (-1.787 | - | 1.126) | 0.656 |
|  | EOS | -11.125 | (-23.525 | - | 1.274) | 0.079 |  | -13.819 | (-24.494 | - | -3.144) | 0.011 |
|  |  |  |  |  |  |  |  |  |  |  |  |  |

A) Adjusted for EOS, NEU, age, height, sex, no of years of follow-up. B) Adjusted for EOS, NEU, age, height, sex, no of years of follow-up, packyears of smoking at follow-up (among ever-smokers), occupational exposure to VGDF at follow-up. C) Adjusted for EOS, NEU, age, height, sex, no of years of follow-up, packyears of smoking at follow-up (among ever-smokers), occupational exposure to VGDF at follow-up, BMI, allergic sensitization. D) Adjusted for EOS, NEU, age, height, sex, no of years of follow-up, packyears of smoking at follow-up (among ever-smokers), occupational exposure to VGDF at follow-up, BMI, allergic sensitization, plus ICS use and OCS use, respectively, at both study entry and follow-up, FEV1<80% at study entry, and positive reversibility at study entry. Green color indicates statistical significance.

**Table E3. Mean annual change in FEV1% of predicted by one unit change in absolute levels (1.0*10^9^/L) of eosinophils (EOS) and neutrophils (NEU) in blood, assessed by B-coefficients with 95% CI and corresponding p-values from linear regression models, among women and men, respectively**

|  |  | **Among women (n=495)** | | | | |  | **Among men (n=397)** | | | | |
| --- | --- | --- | --- | --- | --- | --- | --- | --- | --- | --- | --- | --- |
|  |  | **B** | **(95% CI)** | | | **P-value** |  | **B** | **(95% CI)** | | | **P-value** |
|  |  |  |  |  |  |  |  |  |  |  |  |  |
| Unadjusted | NEU | -0.025 | (-0.061 | - | 0.011) | 0.167 |  | -0.078 | (-0.124 | - | -0.031) | 0.001 |
|  | EOS | -0.530 | (-0.862 | - | -0.199) | 0.002 |  | -0.222 | (-0.573 | - | 0.130) | 0.215 |
|  |  |  |  |  |  |  |  |  |  |  |  |  |
| Adjusted A) | NEU | -0.018 | (-0.053 | - | 0.017) | 0.307 |  | -0.079 | (-0.127 | - | -0.032) | 0.001 |
|  | EOS | -0.597 | (-0.923 | - | -0.272) | <0.001 |  | -0.194 | (-0.551 | - | 0.163) | 0.286 |
|  |  |  |  |  |  |  |  |  |  |  |  |  |
| Adjusted B) | NEU | -0.008 | (-0.044 | - | 0.027) | 0.640 |  | -0.060 | (-0.108 | - | -0.012) | 0.015 |
|  | EOS | -0.550 | (-0.880 | - | -0.220) | 0.001 |  | -0.288 | (-0.645 | - | 0.070) | 0.115 |
|  |  |  |  |  |  |  |  |  |  |  |  |  |
| Adjusted C) | NEU | -0.004 | (-0.040 | - | 0.032) | 0.828 |  | -0.057 | (-0.106 | - | -0.009) | 0.021 |
|  | EOS | -0.512 | (-0.847 | - | -0.177) | 0.003 |  | -0.302 | (-0.666 | - | 0.061) | 0.103 |
|  |  |  |  |  |  |  |  |  |  |  |  |  |
| Adjusted D) | NEU | -0.003 | (-0.039 | - | 0.033) | 0.859 |  | -0.050 | (-0.098 | - | -0.002) | 0.040 |
|  | EOS | -0.548 | (-0.887 | - | -0.209) | 0.002 |  | -0.280 | (-0.637 | - | 0.078) | 0.125 |
|  |  |  |  |  |  |  |  |  |  |  |  |  |

A) Adjusted for EOS, NEU, age, height, no of years of follow-up. B) Adjusted for EOS, NEU, age, height, no of years of follow-up, packyears of smoking at follow-up, occupational exposure to VGDF at follow-up. C) Adjusted for EOS, NEU, age, height, no of years of follow-up, packyears of smoking at follow-up, occupational exposure to VGDF at follow-up, BMI, allergic sensitization. D) Adjusted for EOS, NEU, age, height, no of years of follow-up, packyears of smoking at follow-up, occupational exposure to VGDF at follow-up, BMI, allergic sensitization, plus ICS use and OCS use, respectively, at both study entry and follow-up, FEV1<80% at study entry, and positive reversibility at study entry. Green color indicates statistical significance.

**Table E4. Mean annual change in FEV1ml by one unit change in absolute levels (1.0*10^9^/L) of eosinophils (EOS) and neutrophils (NEU) in blood, assessed by B-coefficients with 95% CI and corresponding p-values from linear regression models, among women and men, respectively**

|  |  | **Among women (n=495)** | | | | |  | **Among men (n=397)** | | | | |
| --- | --- | --- | --- | --- | --- | --- | --- | --- | --- | --- | --- | --- |
|  |  | **B** | **(95% CI)** | | | **P-value** |  | **B** | **(95% CI)** | | | **P-value** |
|  |  |  |  |  |  |  |  |  |  |  |  |  |
| Unadjusted | NEU | -0.090 | (-1.092 | - | 0.912) | 0.860 |  | -3.038 | (-4.928 | - | -1.148) | 0.002 |
|  | EOS | -15.843 | (-25.090 | - | -6.595) | 0.001 |  | -10.058 | (-24.438 | - | 4.321) | 0.170 |
|  |  |  |  |  |  |  |  |  |  |  |  |  |
| Adjusted A) | NEU | -0.262 | (-1.228 | - | 0.704) | 0.594 |  | -2.863 | (-4.775 | - | -0.952) | 0.003 |
|  | EOS | -13.713 | (-22.713 | - | -4.712) | 0.003 |  | -5.760 | (-20.193 | - | 8.674) | 0.433 |
|  |  |  |  |  |  |  |  |  |  |  |  |  |
| Adjusted B) | NEU | 0.061 | (-0.925 | - | 1.047) | 0.903 |  | -2.219 | (-4.173 | - | -0.264) | 0.026 |
|  | EOS | -13.436 | (-22.599 | - | -4.273) | 0.004 |  | -9.238 | (-23.794 | - | 5.319) | 0.213 |
|  |  |  |  |  |  |  |  |  |  |  |  |  |
| Adjusted C) | NEU | 0.239 | (-0.755 | - | 1.233) | 0.637 |  | -2.127 | (-4.104 | - | -0.149) | 0.035 |
|  | EOS | -12.171 | (-21.446 | - | -2.895) | 0.010 |  | -9.685 | (-24.458 | - | 5.089) | 0.198 |
|  |  |  |  |  |  |  |  |  |  |  |  |  |
| Adjusted D) | NEU | 0.212 | (-0.765 | - | 1.189) | 0.669 |  | -1.795 | (-3.675 | - | 0.086) | 0.061 |
|  | EOS | -14.621 | (-23.838 | - | -5.404) | 0.002 |  | -9.756 | (-23.854 | - | 4.341) | 0.174 |
|  |  |  |  |  |  |  |  |  |  |  |  |  |

A) Adjusted for EOS, NEU, age, height, no of years of follow-up. B) Adjusted for EOS, NEU, age, height, no of years of follow-up, packyears of smoking at follow-up, occupational exposure to VGDF at follow-up. C) Adjusted for EOS, NEU, age, height, no of years of follow-up, packyears of smoking at follow-up, occupational exposure to VGDF at follow-up, BMI, allergic sensitization. D) Adjusted for EOS, NEU, age, height, no of years of follow-up, packyears of smoking at follow-up, occupational exposure to VGDF at follow-up, BMI, allergic sensitization, plus ICS use and OCS use, respectively, at both study entry and follow-up, FEV1<80% at study entry, and positive reversibility at study entry. Green color indicates statistical significance.

**Table E5. Mean annual change in FEV1% of predicted and FEV1ml, respectively, by one unit change in absolute levels of eosinophils (EOS) and neutrophils (NEU) in blood, assessed by B-coefficients with 95% CI and corresponding p-values from linear regression models with EOS and NEU entered as log-transformed variables into the models**

|  |  | **With FEV1% of predicted as outcome** | | | | |  | **With FEV1ml as outcome** | | | | |
| --- | --- | --- | --- | --- | --- | --- | --- | --- | --- | --- | --- | --- |
|  |  | **B** | **(95% CI)** | | | **P-value** |  | **B** | **(95% CI)** | | | **P-value** |
|  |  |  |  |  |  |  |  |  |  |  |  |  |
| Unadjusted | NEU | -0.442 | (-0.708 | - | 0.324) | 0.001 |  | -11.352 | (-20.608 | - | -2.096) | 0.016 |
|  | EOS | -0.221 | (-0.345 | - | -0.097) | 0.001 |  | -8.139 | (-12.454 | - | -3.823) | <0.001 |
|  |  |  |  |  |  |  |  |  |  |  |  |  |
| Adjusted A) | NEU | -0.448 | (-0.710 | - | -0.186) | 0.001 |  | -12.135 | (-21.084 | - | -3.185) | 0.008 |
|  | EOS | -0.198 | (-0.322 | - | -0.073) | 0.002 |  | -5.052 | (-9.303 | - | -0.800) | 0.020 |
|  |  |  |  |  |  |  |  |  |  |  |  |  |
| Adjusted B) | NEU | -0.247 | (-0.513 | - | 0.019) | 0.069 |  | -5.883 | (-15.055 | - | 3.289) | 0.208 |
|  | EOS | -0.204 | (-0.327 | - | -0.080) | 0.001 |  | -5.634 | (-9.898 | - | -1.370) | 0.010 |
|  |  |  |  |  |  |  |  |  |  |  |  |  |
| Adjusted C) | NEU | -0.203 | (-0.472 | - | 0.065) | 0.138 |  | -3.854 | (-13.095 | - | 5.386) | 0.413 |
|  | EOS | -0.196 | (-0.321 | - | -0.070) | 0.002 |  | -5.326 | (-9.628 | - | -1.023) | 0.015 |
|  |  |  |  |  |  |  |  |  |  |  |  |  |
| Adjusted D) | NEU | -0.168 | (-0.435 | - | 0.099) | 0.218 |  | -2.546 | (-11.443 | - | 6.350) | 0.574 |
|  | EOS | -0.192 | (-0.317 | - | -0.068) | 0.003 |  | -5.684 | (-9.843 | - | -1.525) | 0.007 |
|  |  |  |  |  |  |  |  |  |  |  |  |  |

A) Adjusted for EOS, NEU, age, height, no of years of follow-up. B) Adjusted for EOS, NEU, age, height, no of years of follow-up, packyears of smoking at follow-up, occupational exposure to VGDF at follow-up. C) Adjusted for EOS, NEU, age, height, no of years of follow-up, packyears of smoking at follow-up, occupational exposure to VGDF at follow-up, BMI, allergic sensitization. D) Adjusted for EOS, NEU, age, height, no of years of follow-up, packyears of smoking at follow-up, occupational exposure to VGDF at follow-up, BMI, allergic sensitization, plus ICS use and OCS use, respectively, at both study entry and follow-up, FEV1<80% at study entry, and positive reversibility at study entry. Green color indicates statistical significance. EOS and NEU Log10-transformed in all models.

**Table E6. Odds ratios (OR) with 95% CI for the association between more rapid decline in FEV1 % of predicted and subjects with higher absolute levels (1.0*10^9^/L) of eosinophils (EOS) in blood, with EOS<0.3 as reference**

|  |  |  |  |  | **Categories of eosinophils in blood as covariates** | | | | | | | | |
| --- | --- | --- | --- | --- | --- | --- | --- | --- | --- | --- | --- | --- | --- |
|  |  |  |  |  | **0.3≤EOS<0.4** | | | |  | **EOS≥0.4** | | | |
|  | **Outcome variable FEV1 % of predicted** | | |  |  |  |  |  |  |  |  |  |  |
|  | **"Worst" FEV1 decline as defined below** | | |  | **OR** | **(95% CI)** | | |  | **OR** | **(95% CI)** | | |
|  |  |  |  |  |  |  |  |  |  |  |  |  |  |
|  | Tertile | 33% lowest values | (n=297) | | 1.4 | (0.9 | - | 2.1) |  | 2.0 | (1.3 | - | 2.9) |
|  | Quartile | 25% lowest values | (n=223) | | 1.5 | (0.96 | - | 2.3) |  | 2.4 | (1.6 | - | 3.6) |
| Unadjusted | Quintile | 20% lowest values | (n=178) | | 1.6 | (1.03 | - | 2.6) |  | 2.5 | (1.7 | - | 3.9) |
|  | Sextile | 17% lowest values | (n=148) | | 1.9 | (1.2 | - | 3.1) |  | 2.4 | (1.5 | - | 3.8) |
|  | Septile | 14% lowest values | (n=127) | | 1.9 | (1.1 | - | 3.2) |  | 2.8 | (1.7 | - | 4.4) |
|  |  |  |  |  |  |  |  |  |  |  |  |  |  |
|  | Tertile | 33% lowest values | (n=297) | | 1.4 | (0.9 | - | 2.2) |  | 1.9 | (1.3 | - | 2.8) |
|  | Quartile | 25% lowest values | (n=223) | | 1.5 | (0.9 | - | 2.3) |  | 2.3 | (1.5 | - | 3.5) |
| Adjusted | Quintile | 20% lowest values | (n=178) | | 1.6 | (0.97 | - | 2.6) |  | 2.4 | (1.5 | - | 3.7) |
|  | Sextile | 17% lowest values | (n=148) | | 1.9 | (1.1 | - | 3.1) |  | 2.2 | (1.4 | - | 3.6) |
|  | Septile | 14% lowest values | (n=127) | | 1.9 | (1.1 | - | 3.3) |  | 2.6 | (1.6 | - | 4.3) |
|  |  |  |  |  |  |  |  |  |  |  |  |  |  |

Adjusted = adjusted by logistic regression models with categories of NEU (4.0≤NEU<5.0 and NEU≥5.0, respectively, compared to NEU<4.0 as reference), age, height, sex, no of years of follow-up, packyears of smoking at follow-up, occupational exposure to VGDF at follow-up, BMI, allergic sensitization, ICS use and OCS use, respectively, at both study entry and follow-up, FEV1<80% at study entry, and positive reversibility at study entry as covariates.
Green color indicates statistical significance.

**Table E7. Odds ratios (OR) with 95% CI for the association between more rapid decline in FEV1ml and subjects with higher absolute levels (1.0*10^9^/L) of eosinophils (EOS) in blood, with EOS<0.3 as reference**

|  |  |  |  |  | **Categories of eosinophils in blood as covariates** | | | | | | | | |
| --- | --- | --- | --- | --- | --- | --- | --- | --- | --- | --- | --- | --- | --- |
|  |  |  |  |  | **0.3≤EOS<0.4** | | | |  | **EOS≥0.4** | | | |
|  | **Outcome variable FEV1ml** | |  |  |  |  |  |  |  |  |  |  |  |
|  | **"Worst" FEV1 decline as defined below** | | |  | **OR** | **(95% CI** | | |  | **OR** | **(95% CI)** | | |
|  |  |  |  |  |  |  |  |  |  |  |  |  |  |
|  | Tertile | 33% lowest values | (n=297) | | 1.4 | (0.9 | - | 2.1) |  | 1.7 | (1.2 | - | 2.6) |
|  | Quartile | 25% lowest values | (n=223) | | 1.5 | (0.97 | - | 2.3) |  | 2.0 | (1.3 | - | 2.9) |
| Unadjusted | Quintile | 20% lowest values | (n=178) | | 1.3 | (0.8 | - | 2.1) |  | 1.9 | (1.3 | - | 3.0) |
|  | Sextile | 17% lowest values | (n=148) | | 1.5 | (0.9 | - | 2.5) |  | 2.3 | (1.5 | - | 3.6) |
|  | Septile | 14% lowest values | (n=127) | | 1.4 | (0.8 | - | 2.5) |  | 2.6 | (1.6 | - | 4.1) |
|  |  |  |  |  |  |  |  |  |  |  |  |  |  |
|  | Tertile | 33% lowest values | (n=297) | | 1.3 | (0.8 | - | 2.0) |  | 1.5 | (0.9 | - | 2.2) |
|  | Quartile | 25% lowest values | (n=223) | | 1.4 | (0.9 | - | 2.2) |  | 1.6 | (1.02 | - | 2.5) |
| Adjusted | Quintile | 20% lowest values | (n=178) | | 1.2 | (0.7 | - | 2.0) |  | 1.6 | (0.97 | - | 2.5) |
|  | Sextile | 17% lowest values | (n=148) | | 1.4 | (0.8 | - | 2.4) |  | 1.9 | (1.1 | - | 3.0) |
|  | Septile | 14% lowest values | (n=127) | | 1.3 | (0.7 | - | 2.4) |  | 2.2 | (1.3 | - | 3.6) |
|  |  |  |  |  |  |  |  |  |  |  |  |  |  |

Adjusted = adjusted by logistic regression models with categories of NEU (4.0≤NEU<5.0 and NEU≥5.0, respectively, compared to NEU<4.0 as reference), age, height, sex, no of years of follow-up, packyears of smoking at follow-up, occupational exposure to VGDF at follow-up, BMI, allergic sensitization, ICS use and OCS use, respectively, at both study entry and follow-up, FEV1<80% at study entry, and positive reversibility at study entry as covariates.
Green color indicates statistical significance.

**Table E8. Odds ratios (OR) with 95% CI for the association between more rapid decline in FEV1 Z-score based on the OLIN* reference values for spirometry and subjects with higher absolute levels (1.0*10^9^/L) of eosinophils (EOS) in blood, with EOS<0.3 as reference**

|  |  |  |  |  | **Categories of eosinophils in blood as covariates** | | | | | | | | |
| --- | --- | --- | --- | --- | --- | --- | --- | --- | --- | --- | --- | --- | --- |
|  |  |  |  |  | **0.3≤EOS<0.4** | | | |  | **EOS≥0.4** | | | |
|  | **Outcome variable FEV1 Z-score** | |  |  |  |  |  |  |  |  |  |  |  |
|  | **"Worst" FEV1 decline as defined below** | | |  | **OR** | **(95% CI)** | | |  | **OR** | **(95% CI)** | | |
|  |  |  |  |  |  |  |  |  |  |  |  |  |  |
|  | Tertile | 33% lowest values | (n=297) | | 1.3 | (0.9 | - | 2.0) |  | 2.1 | (1.4 | - | 3.2) |
|  | Quartile | 25% lowest values | (n=222) | | 1.5 | (0.9 | - | 2.3) |  | 2.0 | (1.4 | - | 3.1) |
| Unadjusted | Quintile | 20% lowest values | (n=178) | | 1.6 | (0.99 | - | 2.6) |  | 2.2 | (1.4 | - | 3.4) |
|  | Sextile | 17% lowest values | (n=148) | | 1.9 | (1.2 | - | 3.1) |  | 2.3 | (1.5 | - | 3.6) |
|  | Septile | 14% lowest values | (n=127) | | 1.6 | (0.9 | - | 2.7) |  | 2.4 | (1.5 | - | 3.8) |
|  |  |  |  |  |  |  |  |  |  |  |  |  |  |
|  | Tertile | 33% lowest values | (n=297) | | 1.4 | (0.9 | - | 2.1) |  | 2.1 | (1.4 | - | 3.2) |
|  | Quartile | 25% lowest values | (n=222) | | 1.5 | (0.96 | - | 2.5) |  | 2.2 | (1.4 | - | 3.4) |
| Adjusted | Quintile | 20% lowest values | (n=178) | | 1.7 | (1.01 | - | 2.8) |  | 2.4 | (1.5 | - | 3.8) |
|  | Sextile | 17% lowest values | (n=148) | | 2.0 | (1.2 | - | 3.3) |  | 2.4 | (1.5 | - | 3.8) |
|  | Septile | 14% lowest values | (n=127) | | 1.7 | (0.9 | - | 3.0) |  | 2.5 | (1.5 | - | 4.1) |
|  |  |  |  |  |  |  |  |  |  |  |  |  |  |

Adjusted = adjusted by logistic regression models with categories of NEU (4.0≤NEU<5.0 and NEU≥5.0, respectively, compared to NEU<4.0 as reference), age, height, sex, no of years of follow-up, packyears of smoking at follow-up, occupational exposure to VGDF at follow-up, BMI, allergic sensitization, ICS use and OCS use, respectively, at both study entry and follow-up, FEV1<80% at study entry, and positive reversibility at study entry as covariates.
Green color indicates statistical significance. OLIN = Obstructive Lung Disease in Northern Sweden. Z-score = standardized residual for the OLIN reference value[1].

**Table E9. Odds ratios (OR) with 95% CI for the association between more rapid decline in FEV1 Z-score based on the GLI* reference values for spirometry and subjects with higher absolute levels (1.0*10^9^/L) of eosinophils (EOS) in blood, with EOS<0.3 as reference**

|  |  |  |  |  | **Categories of eosinophils in blood as covariates** | | | | | | | | |
| --- | --- | --- | --- | --- | --- | --- | --- | --- | --- | --- | --- | --- | --- |
|  |  |  |  |  | **0.3≤EOS<0.4** | | | |  | **EOS≥0.4** | | | |
|  | **Outcome variable FEV1 Z-score (GLI)** | | |  |  |  |  |  |  |  |  |  |  |
|  | **"Worst" FEV1 decline as defined below** | | |  | **OR** | **(95% CI)** | | |  | **OR** | **(95% CI)** | | |
|  |  |  |  |  |  |  |  |  |  |  |  |  |  |
|  | Tertile | 33% lowest values | (n=298) | | 1.3 | (0.9 | - | 2.0) |  | 1.9 | (1.3 | - | 2.7) |
|  | Quartile | 25% lowest values | (n=222) | | 1.7 | (1.1 | - | 2.6) |  | 2.1 | (1.4 | - | 3.1) |
| Unadjusted | Quintile | 20% lowest values | (n=178) | | 1.6 | (1.0 | - | 2.6) |  | 2.4 | (1.6 | - | 3.7) |
|  | Sextile | 17% lowest values | (n=148) | | 1.8 | (1.1 | - | 3.0) |  | 2.7 | (1.8 | - | 4.3) |
|  | Septile | 14% lowest values | (n=127) | | 1.7 | (1.0 | - | 2.8) |  | 2.7 | (1.7 | - | 4.2) |
|  |  |  |  |  |  |  |  |  |  |  |  |  |  |
|  | Tertile | 33% lowest values | (n=297) | | 1.4 | (0.9 | - | 2.1) |  | 1.9 | (1.3 | - | 2.9) |
|  | Quartile | 25% lowest values | (n=222) | | 1.7 | (1.1 | - | 2.8) |  | 2.1 | (1.4 | - | 3.3) |
| Adjusted | Quintile | 20% lowest values | (n=178) | | 1.7 | (1.0 | - | 2.8) |  | 2.5 | (1.6 | - | 3.9) |
|  | Sextile | 17% lowest values | (n=148) | | 1.9 | (1.1 | - | 3.2) |  | 2.8 | (1.8 | - | 4.5) |
|  | Septile | 14% lowest values | (n=127) | | 1.7 | (1.0 | - | 3.0) |  | 2.7 | (1.7 | - | 4.5) |
|  |  |  |  |  |  |  |  |  |  |  |  |  |  |

Adjusted = adjusted by logistic regression models with categories of NEU (4.0≤NEU<5.0 and NEU≥5.0, respectively, compared to NEU<4.0 as reference), age, height, sex, no of years of follow-up, packyears of smoking at follow-up, occupational exposure to VGDF at follow-up, BMI, allergic sensitization, ICS use and OCS use, respectively, at both study entry and follow-up, FEV1<80% at study entry, and positive reversibility at study entry as covariates.
Green color indicates statistical significance. *GLI = Global Lung Initiative. Z-score = standardized residual for the GLI reference value[2].

**Table E10. Odds ratios (OR) with 95% CI for the association between more rapid decline in FEV1 % of predicted and subjects with higher absolute levels (1.0*10^9^/L) of eosinophils (EOS) in blood, with EOS<0.15 as reference. Outcome variable was the 25% lowest (“worst”) values assessed by annual decline in FEV1 % of predicted.**

|  |  |  | **Unadjusted** | | | | |  |  | **Adjusted** | | | | |
| --- | --- | --- | --- | --- | --- | --- | --- | --- | --- | --- | --- | --- | --- | --- |
|  |  | **P-value** | **OR** |  | **(95% CI)** | | |  | **P-value** | **OR** |  | **(95% CI)** | | |
|  |  |  |  |  |  |  |  |  |  |  |  |  |  |  |
| EOS<0.15 | Reference (n=355) |  |  |  |  |  |  |  |  |  |  |  |  |  |
| 0.15≤EOS<0.3 | (n=294) | 0.334 | 1.20 |  | (0.83 | - | 1.75) |  | 0.422 | 1.18 |  | (0.79 | - | 1.75) |
| 0.3≤EOS<0.4 | (n=114) | 0.047 | 1.63 |  | (1.01 | - | 2.64) |  | 0.084 | 1.57 |  | (0.94 | - | 2.63) |
| EOS≥0.4 | (n=129) | 0.000 | 2.62 |  | (1.69 | - | 4.05) |  | 0.000 | 2.47 |  | (1.54 | - | 3.96) |
|  |  |  |  |  |  |  |  |  |  |  |  |  |  |  |

Adjusted = adjusted by logistic regression models with categories of NEU (4.0≤NEU<5.0 and NEU≥5.0, respectively, compared to NEU<4.0 as reference), age, height, sex, no of years of follow-up, packyears of smoking at follow-up, occupational exposure to VGDF at follow-up, BMI, allergic sensitization, ICS use and OCS use, respectively, at both study entry and follow-up, FEV1<80% at study entry, and positive reversibility at study entry as covariates.


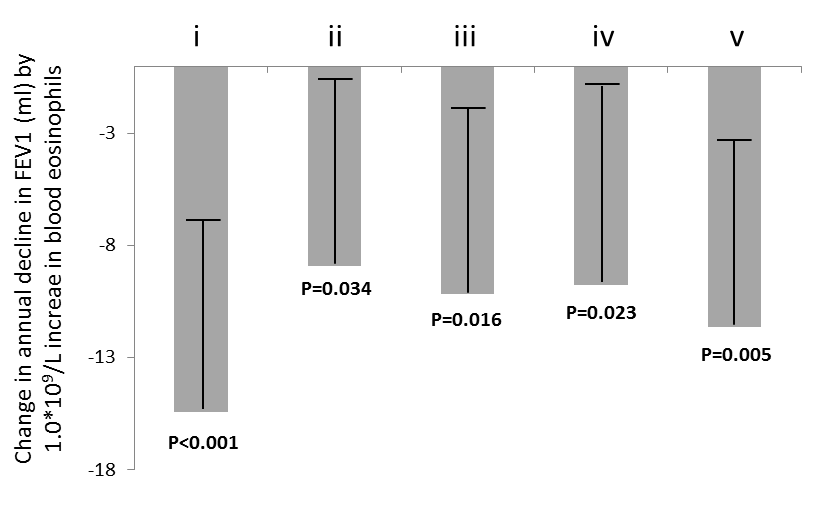


Supplemental Figure E1. Mean annual change in FEV1 measured in ml by one unit change in absolute levels (1.0*10^9^/L) of blood eosinophils at follow-up, assessed by B-coefficients with 95% CI and corresponding p-values from linear regression models. (i)=Unadjusted, (ii)=Adjusted for neutrophils in blood, number of years of follow-up, age, height and sex, (iii)=Adjusted for the same as (ii) and also for number of packyears of smoking and exposure to vapors, gas, dust or fumes at work, (iv)=Adjusted for the same as (iii) but also for BMI and allergic sensitization, (v)=Same as (iv) but also for ICS use and OCS use at study entry and/or follow-up, FEV1<80% of predicted at study entry, and significant FEV1 reversibility at study entry.


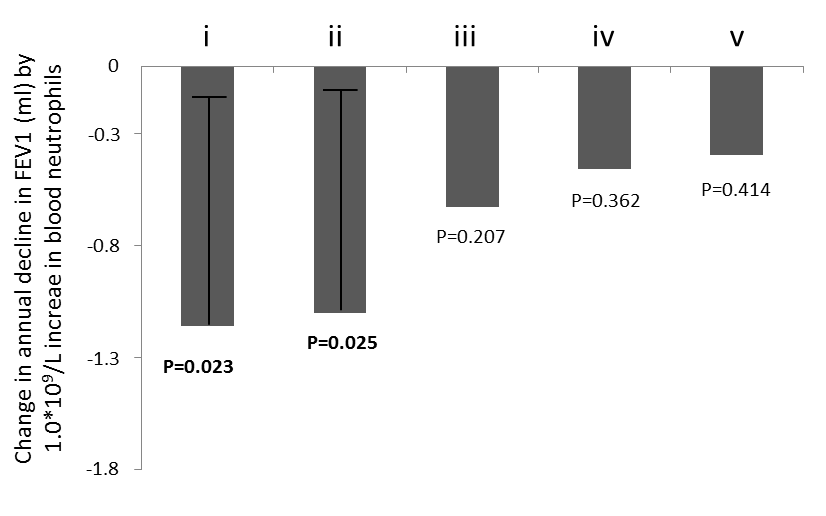


Supplemental Figure E2. Mean annual change in FEV1 measured in ml by one unit change in absolute levels (1.0*10^9^/L) of blood neutrophils at follow-up, assessed by B-coefficients with 95% CI and corresponding p-values from linear regression models. (i)=Unadjusted, (ii)=Adjusted for eosinophils in blood, number of years of follow-up, age, height and sex, (iii)=Adjusted for the same as (ii) and also for number of packyears of smoking and exposure to vapors, gas, dust or fumes at work, (iv)=Adjusted for the same as (iii) but also for BMI and allergic sensitization, (v)=Same as (iv) but also for ICS use and OCS use at study entry and/or follow-up, FEV1<80% of predicted at study entry, and significant FEV1 reversibility at study entry.

**Age at asthma onset in relation to FEV_1_ decline**

In total, when combining information attained at the examinations performed at baseline and follow-up, n=828 of the included individuals had available data on age at asthma onset, whereof n=219 had asthma onset before 16 years of age. Among those with available data on age at asthma onset, the mean annual FEV_1_ decline among those with onset <16 years of age versus ≥16 years of age was -0.09 vs -0.07 (p=0.639) in terms of FEV_1_pp and -25ml vs -28ml (p=0.050) in terms of FEV_1_ml.

**FEV_1_ decline based on post-bronchodilator results**

As post-bronchodilator spirometry results only were available for a subsample at baseline, FEV_1_ decline based on post-bronchodilator data could only be calculated for this subsample. The criterion for performing reversibility testing at baseline was FEV_1_/VC<0.7 and/or FEV_1_<90% of predicted. In contrast, reversibility testing was performed among all subjects in the 2012-2014 follow-up. In total, post-bronchodilator data at both baseline and follow-up was available for n=246. The mean annual decline in FEV_1_ in this subgroup was -24ml based on pre-bronchodilator values compared to -27ml based on post-bronchodilator values. Further, among the n=246 individuals with available post-bronchodilator data, the Spearman correlation coefficients between FEV_1_ decline and blood EOS were -0.124 (p=0.052) based on pre-bronchodilator values, and -0.129 (p=0.043) based on post-bronchodilator values.

**References**

E1. Backman H, Lindberg A, Oden A, Ekerljung L, Hedman L, Kainu A, Sovijärvi A, Lundbäck B, Rönmark E. Reference values for spirometry - report from the Obstructive Lung Disease in Northern Sweden studies. Eur Clin Respir J 2015; 2: 10.3402/ecrj.v2.26375. eCollection 2015.

E2. Quanjer PH, Stanojevic S, Cole TJ, Baur X, Hall GL, Culver BH, Enright PL, Hankinson JL, Ip MS, Zheng J, Stocks J; ERS Global Lung Function Initiative. Multi-ethnic reference values for spirometry for the 3-95-yr age range: the global lung function 2012 equations. Eur Respir J 2012; 40: 1324-43.
