# Supplementary material for: FEV1 decline in relation to blood eosinophils and neutrophils in a population-based asthma cohort
Source: World Allergy Organ J. 2020 Mar 17;13(3):100110. doi: 10.1016/j.waojou.2020.100110 (PMC7082214; doi:10.1016/j.waojou.2020.100110)
Supplement: Multimedia component 2 [file mmc2.pptx]

## Slide 1
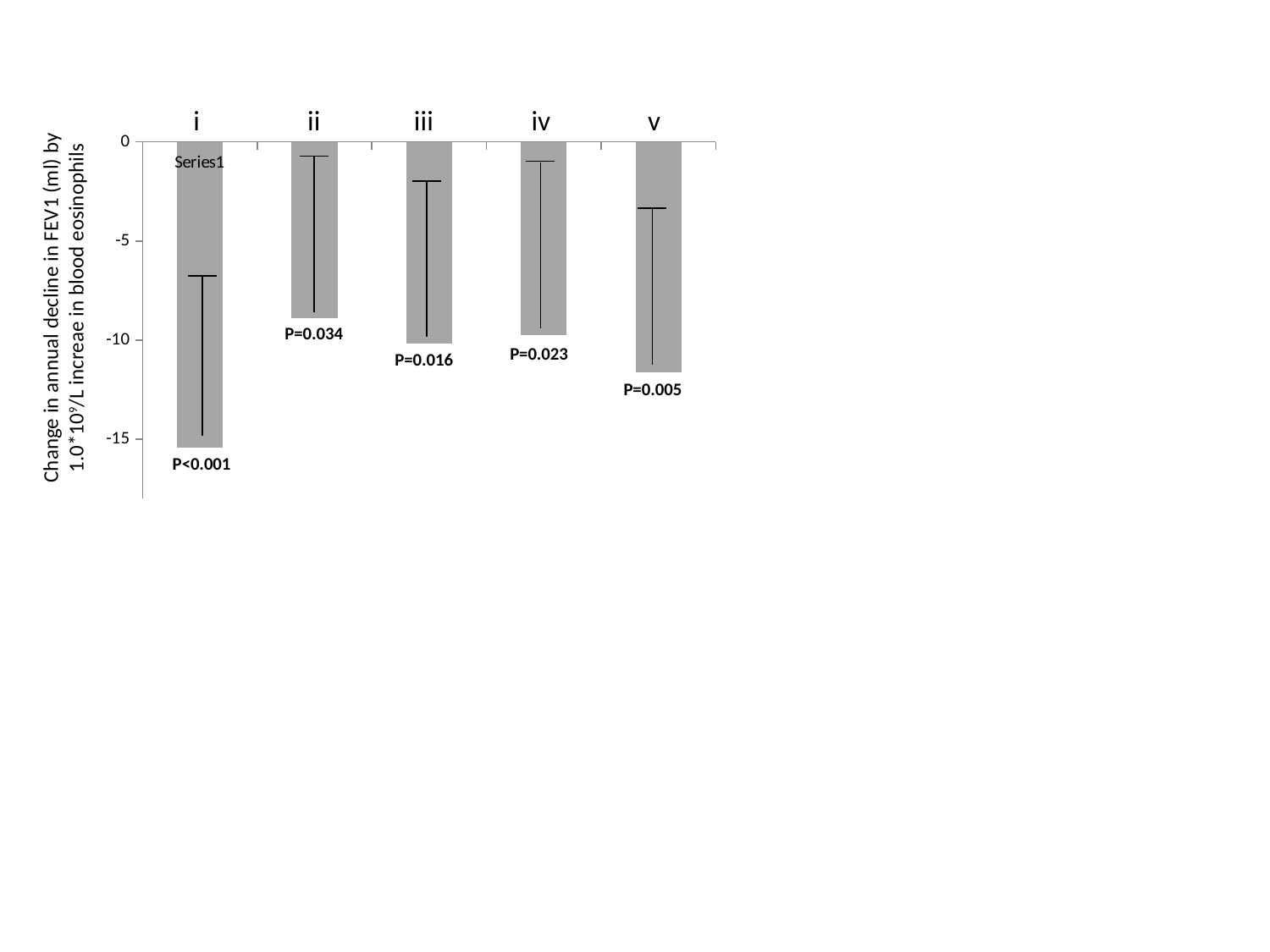

i
ii
iii
iv
v
### Chart
| Category | Mean annual change in FEV1 (ml) |
|---|---|
| | -15.414140249389671 |
| | -8.910841907272605 |
| | -10.170908440128674 |
| | -9.761933124952415 |
| | -11.64441982287789 |P=0.034
P=0.023
P=0.016
P=0.005
P<0.001
Change in annual decline in FEV1 (ml) by 1.0*109/L increae in blood eosinophils
