# Supplementary material for: FEV1 decline in relation to blood eosinophils and neutrophils in a population-based asthma cohort
Source: World Allergy Organ J. 2020 Mar 17;13(3):100110. doi: 10.1016/j.waojou.2020.100110 (PMC7082214; doi:10.1016/j.waojou.2020.100110)
Supplement: Multimedia component 3 [file mmc3.pptx]

## Slide 1
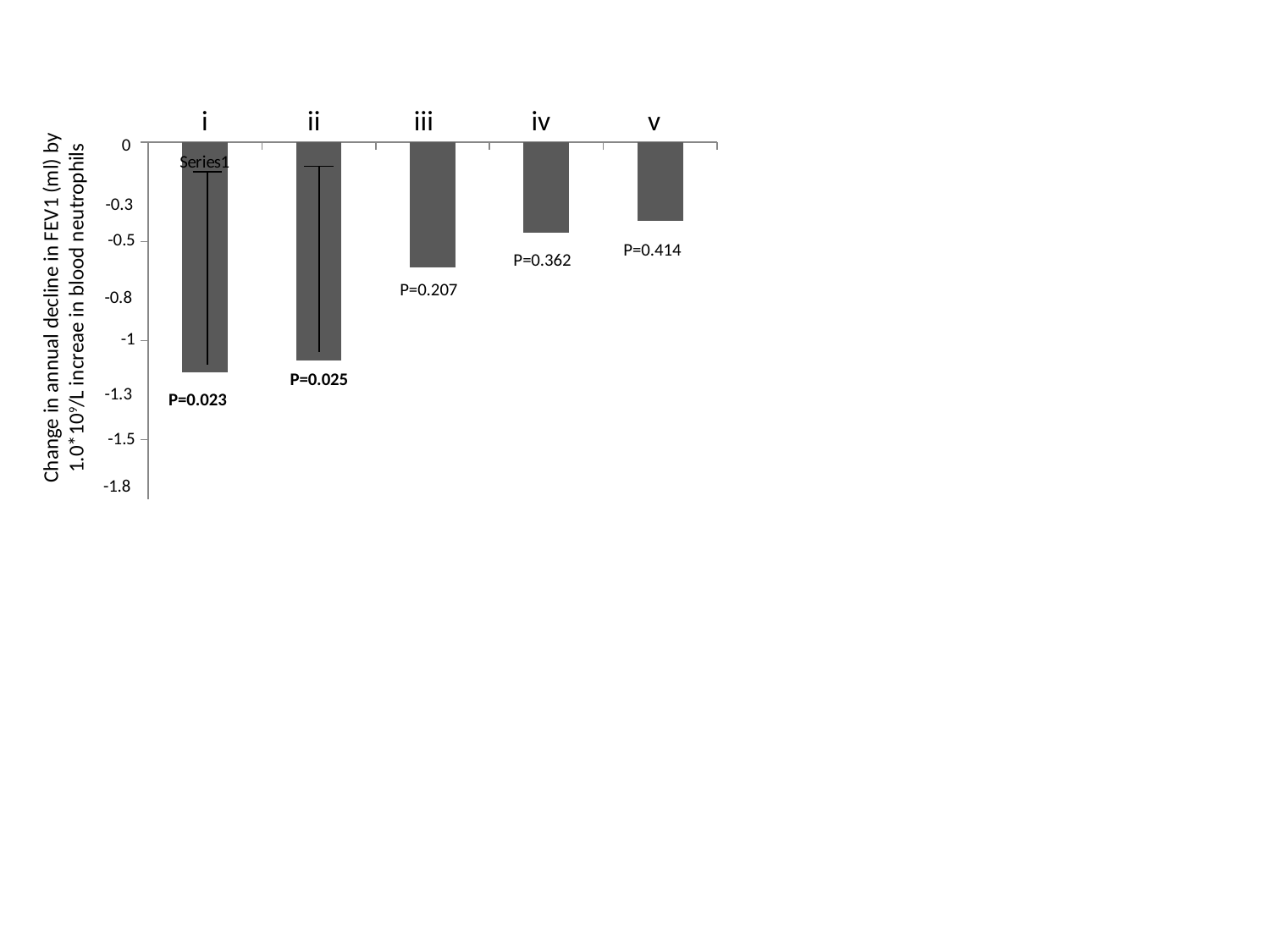

i
ii
iii
iv
v
### Chart
| Category | Mean annual change in FEV1 (ml) |
|---|---|
| | -1.160667818206499 |
| | -1.1016919160524832 |
| | -0.6301148814361472 |
| | -0.45738462657548357 |
| | -0.39478539223397613 |P=0.414
P=0.362
P=0.207
P=0.025
P=0.023
0
-0.3
Change in annual decline in FEV1 (ml) by 1.0*109/L increae in blood neutrophils
-0.8
-1.3
-1.8
